# Value in maternal care: Using the Learning Health System to facilitate action

**DOI:** 10.1111/birt.12684

**Published:** 2022-10-20

**Authors:** Emily J. Callander, Helena Teede, Joanne Enticott

**Affiliations:** ^1^ School of Public Health and Preventive Medicine Monash University Melbourne Victoria Australia; ^2^ Monash Centre for Health Research and Implementation Monash University Melbourne Victoria Australia

**Keywords:** Cost, Pregnancy, Women's Health

## Abstract

There is an increasing need to deliver high‐value health care. Here, we discuss how value should be measured and implemented in maternity care through a Learning Health System. High‐value maternity care will produce the highest level of benefit for women at a given cost. As pregnancy is not an illness state, and there is no cure or remission to be achieved, we believe that patient‐reported outcomes should be an integral component of benefit quantification when measuring value. Furthermore, as care impacts more than just health outcomes—particularly in maternity care—there is also a need to consider patient‐reported experiences as a part of defining the level of benefit. However, to move beyond traditional narrow and passive measurement of value, we need to partner with stakeholders to identify priorities for change, identify evidence for how to achieve this change, integrate measurement activities, and promote effective implementation, in a continuous, learning cycle—a Learning Health System. A robust Framework for implementing a Learning Health System has been developed, which could be applied in maternity care.

During the pandemic, the health care sector has had access to increased resources and expenditure to accommodate the additional demand for care. However, this sector will inevitably face increased austerity moving forward. It is estimated that 30% of health care is waste, duplication, or of low value,^1^ and thus there is the capacity for improvement. Although there has previously been constraints on health service operation expenses,^2^ in moving forward, ensuring *value* in health care will become even more critical in the planning, delivery, monitoring, and improvement of care into the future. Meeting the needs of our community is central to value‐based care, which goes beyond cost to encompass patient and provider experience, quality care and outcomes, efficiency and sustainability.^3^, ^4^ Value‐based care reorients care away from serving the funder, services, provider and system, toward serving community needs, based on evidence and quality.

Achieving value in health care will require a systems‐level transformation from what is currently globally a complex, cumbersome system (with multiple providers of and pathways for care^5^), to being more adaptive, and dynamic. Ongoing change will be required, with lower value care being replaced by higher value care. The optimal strategy to deliver value driven change is not yet established; however, the Learning Health System^4^ has gained significant traction and evidence is emerging on this approach to drive quality sustainable health system transformation that meets community needs. Evidence from stakeholders, community, and research as well as data driven approaches and health care improvement initiatives, can be iteratively applied and integrated into health care to address impending challenges ahead.

Here, we explore value in health care and how it can be measured, and then introduce the concept of the Learning Health System and explain its role in driving value in maternity care. We define maternity care, as the care provided during pregnancy, intrapartum and up to around 6 weeks postnatally (although this follow‐up postnatal period will differ depending on local health service policies). Although there are many areas of health that may benefit from a Learning Health System approach in delivering value‐based health care, maternity care is an important area with significant potential for transformation.^6^ Maternity care is primarily provided through an acute health service model focused on treating health issues as they arise as opposed to a preventive or primary care model, with most high‐income countries and many low‐ to middle‐income countries transitioning from high to low mortality as obstetric complications are treated and from direct to indirect causes of mortality and morbidity as treatments are leading to longer term, unintended complications.^7^ There is thus a need to monitor a wide range of outcomes that are produced, as well as costs, which are variable across services and jurisdictions.^8^ The nature of maternity care, with a finite time period for each episode, and the availability of routine data,^9^ makes value monitoring highly feasible. We use the term “woman” throughout. This should be taken to include people who do not identify as women but are pregnant or have given birth.^10^

## 1 WHAT IS VALUE IN HEALTH CARE?

Simply put, “value” equates to derived *benefit*. Whether something is good value relates to the valence of that benefit, relative to cost.^11^ High‐value health care occurs when benefit outweighs cost, whereas low‐value health care produces little benefit relative to cost. Quantifying value involves dividing the outcomes by the cost of providing care.^11^ There is also an important distinction between cost‐effectiveness analysis and studies that assess the value of health care. Cost‐effectiveness analysis is focused on the *potential* of a discrete intervention to produce a certain level of outcomes at a certain cost *relative* to another intervention. Measuring the value of health care requires measuring the real‐world overall provision of care and takes a broad view of benefit.

Health care impacts more than just health outcomes and there is a risk that only considering health may overlook some of the wider benefits (or adverse implications) of care. In maternity care, pregnancy is not an illness state, and care focuses on identifying risk, screening, and prevention to limit adverse outcomes. Here, patient‐reported outcomes are central, and measuring a woman's experience of care is essential for identifying the benefits from maternal health services. Experience covers what happened during care and how it happened, including relational elements of care.^12^ Value measurement in maternity care must be founded on both outcomes and experience when quantifying benefit.^13^ This is in line with the Institute for Health care Improvement's Triple Aim,^14^ which includes a focus on health *care* experience, as well as health outcomes and costs.

## 2 HOW TO MEASURE OUTCOMES AND EXPERIENCE IN HEALTH CARE?

The collection of health service use and cost data in health care is relatively well established as a part of reporting for activity‐based funding of health services. Greater complexity lies in the measurement of outcomes. There is an increasing body of literature focused on defining core outcome sets for maternal health care.^15^ Core outcome sets are a consensus‐based agreed minimum set of outcomes that should be measured and reported in all studies and are coproduced with broad stakeholders including community members. These are often developed for the purpose of measuring endpoints and efficacy of interventions in clinical trials. The content of core outcome sets for maternal health care focus on mortality and morbidity, and health indicators such as stillbirth, birthweight, and gestation at birth.^15^ However, many of these factors are not actual health outcomes, and they often do not offer a means for women to express what they value in the experience of their preferred outcome of care.

Ultimately, end‐users are ideally placed to identify the benefits they derive from their care—this includes both outcomes and experiences. The concept of women as a prime informant for the benefit of maternity care is aligned with Patient Reported Outcome Measures (PROMs), and Patient Reported Experience Measures (PREMs), making these factors key when measuring value. However, care must be taken in the construction of such measures, as variation is likely to be seen between individual women, between populations and geographical areas, influenced by cultural and social norms.^16^ In addition, it is important that in the construction of such measures, researchers and clinicians do not impart their own preferences in the development process—women may be asked to report on their individual outcomes and experiences, but if the elements of care that they prefer are not a part of a process, the benefit they value and derive from the care will not be adequately captured. Women are thus informants of not only what *level* of benefit they derived from their care but also what the benefit should be.

The notion of women as a key informant of benefits of care, contrasts with the traditionally held view of the health provider or health researcher determining measurable outcomes. Inclusion of PREMs and PROMs also represents a vital power shift away from women having to fit what the system provides, to the system as a social institution^16^ responsible for accommodating what women as end‐users (and indeed as ultimate funders) prefer and how they are integral to measurement of the value of maternity care. PROMs and PREMs then appear ideal for combination with established cost measurement activities to together measure value in maternity care.

## 3 HOW TO IMPLEMENT VALUE, THE ROLE OF THE LEARNING HEALTH SYSTEM

Simply measuring value, however, is unlikely to be sufficient to drive improvement of value, with current estimates of value in maternity care failing to show improvement over time.^8^ To move beyond passive measurement of value, we need to ascertain consumer and clinician identified priorities for change, integrate measurement activities, identify safe, evidence‐based improvement options, and feed this back into a system level approach for improving high‐quality, high‐value, sustainable care that meets the needs of women. This is a Learning Health System (LHS) approach to value improvement. The LHS is an approach to health care provision that involves capturing routinely collected health data and converting it in a timely fashion into useful information to inform decision making, with the ultimate goal of quality improvement in health care for better health outcomes.^4^ To date, there are only a handful of examples of LHS, with none in maternity care.^4^

**FIGURE 1:**
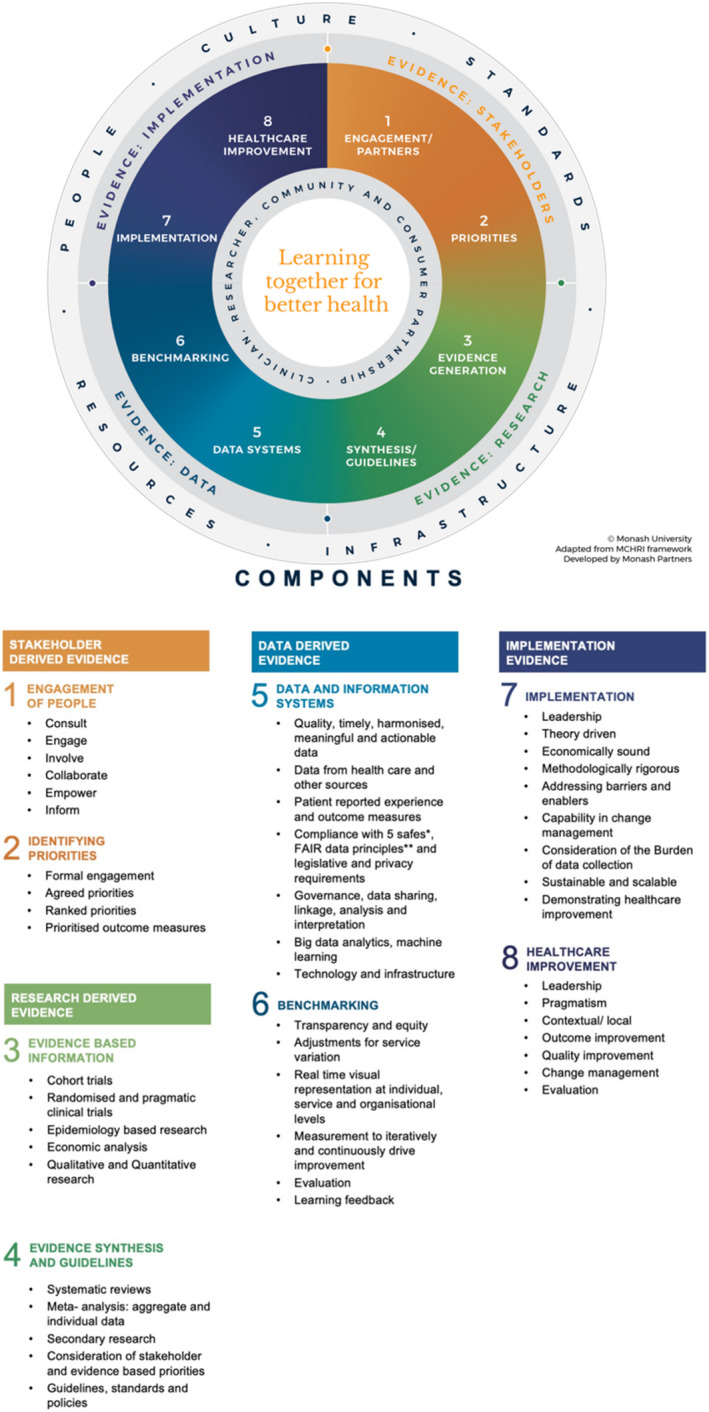
The Monash Learning Health System framework [Color figure can be viewed at wileyonlinelibrary.com]

A LHS approach can be implemented through a new Framework that outlines a robust, codeveloped process.^17^ This framework was co‐produced with all relevant stakeholders, with a diverse steering committee (including community), a systematic review of effective LHS models, extensive qualitative interviews, and coproduction workshops. The LHS Framework has four fundamental evidence pillars to facilitate the LHS process: evidence from stakeholders (community and health professionals, including PREMS and PROMs), evidence from research (including evidence based practice), evidence from routine data on health care performance, and evidence from implementation and improvement to meet stakeholder and community priorities (Figure 1).^17^ The four pillars of evidence come together through an iterative process to drive improvement.

As outlined in Figure 1, initially in Quadrant 1, stakeholders are engaged through information exchange, consultation, and collaboration; and priorities are then agreed on and ranked (including the desired outcomes). Within maternal health care, this could include the co‐identification of women's priority areas for systems change; and the operationalization of women's voices in data collection through the identification and collection of PREMs and PROMs.^18^ As an illustrative example, this may include addressing rising cesarean section rates, which has been previously identified as a priority area.^19^, ^20^ In Quadrant 2, relevant research to support these stakeholder‐identified priorities can then be identified by systematic review of research evidence from clinical trials, epidemiology and health economic studies, qualitative and quantitative research. The evidence can then be synthesized through meta‐analysis, and the formation of guidelines and standards and policies. Previous review articles have identified evidence that demonstrates health system interventions that are effective in reducing cesarean section.^21^ In Quadrant 3, data and information systems can also be used, mostly from routine health care sources, and ideally including PROMS and PREMS, here for timely extraction and analysis with new technologies such as machine learning; these data are then used for benchmarking through real‐time visualization of current performance, with the goal of timely feedback of data analysis to stakeholders (consumers, clinicians, managers) to enable iterative learning from data. Such data can be used to monitor rates of cesarean section, identifying high‐risk groups, variation between health services, outcomes, and cost‐effectiveness of implementation of research evidence from Quadrant 3.^8^, ^22^, ^23^, ^24^ Finally, in Quadrant 4, the stakeholder, research and data evidence can then be combined for implementation through methodologically rigorous and theoretical driven processes to drive health care improvement. This is an iterative ongoing process of learning for health care and outcome improvement.

The Learning Health System Framework differs from other quality improvement and consensus‐based approaches to health systems change, as it combines evidence from four diverse sources—consumers and stakeholders; research; data; and implementation—to remove siloes. It is a quality improvement approach but provides specific guidance on *how* this can be achieved. Often quality improvement initiatives will not involve adequate stakeholder engagement and thus at time of implementation may face barriers as what they are seeking to change does not align with end‐user or consumer priorities.^10^ In the maternity space, such approaches fail to identify that women must be at the front and center of any system change or redesign to truly produce value based health care.

The Learning Health System Framework, centralized around the principle of all stakeholders including end‐users *learning together for better health*, presents an evidence‐based, system level approach to embed routine measurement of value as determined by end‐users. By identifying consumer priorities and designing system change to ensure health care meets the needs of consumers; identifying evidence‐based opportunities to meet these needs to ensure implementation of effective change; using data to identify costs and outcomes, to ensure cost‐effectiveness of change; and using implementation science to ensure timely and sustainable systems change the Learning Health System Framework can help achieve value based care. We believe that such an approach is critical in the delivery of an end‐user focused high‐quality sustainable maternal health system, which is in turn essential for high‐value maternity care and propose a call to arms around approaches to embed end‐users in health system improvement processes. Indeed, this LHS framework is being implemented across health care services and governments, and could be implemented in maternity care and includes co‐development of PREMS and PROMS and embedded assessment of value.

## FUNDING INFORMATION

EC and HT receive salary support from the National Health and Medical Research Council through Fellowship schemes. No further funds were received by the authors for this work.

## Data Availability

Data sharing not applicable to this article as no datasets were generated or analysed during the current study.
